# Wnt-GSK3*β*/*β*-Catenin Regulates the Differentiation of Dental Pulp Stem Cells into Bladder Smooth Muscle Cells

**DOI:** 10.1155/2019/8907570

**Published:** 2019-01-28

**Authors:** Wenkai Jiang, Diya Wang, Amr Alraies, Qian Liu, Bangfu Zhu, Alastair J. Sloan, Longxing Ni, Bing Song

**Affiliations:** ^1^State Key Laboratory of Military Stomatology & National Clinical Research Center for Oral Diseases & Shaanxi Key Laboratory of Stomatology, Department of Operative Dentistry & Endodontics, School of Stomatology, Fourth Military Medical University, No. 145 Western Changle Road, Xi'an, Shaanxi 710032, China; ^2^School of Dentistry, Cardiff Institute of Tissue Engineering and Repair, Cardiff University, Heath Park, Cardiff CF14 4XY, UK; ^3^Department of Occupational and Environmental Health and the Ministry of Education Key Lab of Hazard Assessment and Control in Special Operational Environment, School of Public Health, Fourth Military Medical University, Xi'an, China; ^4^Department of Neurology, Jinling Hospital, School of Medicine, Nanjing University, Nanjing, 210002 Jiangsu Province, China; ^5^School of Biochemistry, University of Bristol, University Walk, Bristol BS8 1TD, UK

## Abstract

Smooth muscle cell- (SMC-) based tissue engineering provides a promising therapeutic strategy for SMC-related disorders. It has been demonstrated that human dental pulp stem cells (DPSCs) possess the potential to differentiate into mature bladder SMCs by induction with condition medium (CM) from bladder SMC culture, in combination with the transforming growth factor-*β*1 (TGF-*β*1). However, the molecular mechanism of SMC differentiation from DPSCs has not been fully uncovered. The canonical Wnt signaling (also known as Wnt/*β*-catenin) pathway plays an essential role in stem cell fate decision. The aim of this study is to explore the regulation via GSK3*β* and associated downstream effectors for SMC differentiation from DPSCs. We characterized one of our DPSC clones with the best proliferation and differentiation abilities. This stem cell clone has shown the capacity to generate a smooth muscle layer-like phenotype after an extended differentiation duration using the SMC induction protocol we established before. We further found that Wnt-GSK3*β*/*β*-catenin signaling is involved in the process of SMC differentiation from DPSCs, as well as a serial of growth factors, including TGF-*β*1, basic fibroblast growth factor (bFGF), epidermal growth factor (EGF), hepatocyte growth factor (HGF), platelet-derived growth factor-homodimer polypeptide of B chain (BB) (PDGF-BB), and vascular endothelial growth factor (VEGF). Pharmacological inhibition on the canonical Wnt-GSK3*β*/*β*-catenin pathway significantly downregulated GSK3*β* phosphorylation and *β*-catenin activation, which in consequence reduced the augmented expression of the growth factors (including TGF-*β*1, HGF, PDGF-BB, and VEGF) as well as SMC markers (especially myosin) at a late stage of SMC differentiation. These results suggest that the canonical Wnt-GSK3*β*/*β*-catenin pathway contributes to DPSC differentiation into mature SMCs through the coordination of different growth factors.

## 1. Introduction

A range of injuries or diseases including cancer, benign bladder contracture, and congenital anomalies (such as bladder exstrophy and myelomeningocele) can result in the damage or loss of the bladder [1–3]. Consequently, patients with those illnesses require bladder augmentation cystoplasty or replacement. However, current cystoplasty using gastrointestinal segments cannot completely restore the normal function of the Detrusor muscle, in some cases even leading to complications, such as metabolic disturbances, urolithiasis, infection, and malignant diseases [4, 5]. Therefore, bladder smooth muscle cell-based tissue engineering becomes one of the most promising remedies for restoring bladder organ function of the urinary system [6, 7]. However, due to the limited expansion of bladder tissue-derived SMCs and highly potential oncogenic risk, the progress of bladder tissue engineering is hampered by lack of a stable SMC source [8, 9]. Thus, search for an alternative cell source is essential for bladder tissue engineering, which could provide a new way to overcome the shortcomings of the aforementioned methods in the future.

Mesenchymal stem cells (MSCs) are one type of fibroblast-like cell population with potential extensive self-renewal and multilineage differentiation abilities [10]. Compared with other MSCs derived from the bone marrow [11], adipose tissue [12], peripheral blood [13], and umbilical cord blood [14], MSCs derived from dental pulp tissues have marked advantages of easy access with the least invasive procedures and capacities of high proliferation, excellent regeneration, and multiple potential of differentiation along with little inherent immunogenicity, which make them particularly suitable for tissue engineering and gene therapy applications [15, 16]. Dental pulp stem cells (DPSCs) are normally isolated using a single colony method as DPSCs from pulp tissue are heterogeneous, which are expected to produce complex biological activities [17, 18]. For this reason, different clones of DPSCs exhibit different behaviours including various capacities of proliferation and differentiation according to the age of the donor, method of isolation, and conditions of the pulp tissue [19]. DPSCs provide a potential source of progenitor cells for tissue engineering. However, the abilities of expansion and differentiation *in vitro* should be fully explored before use. In this study, we have isolated three DPSC clones from different patients. The clones were investigated by comparing their proliferation rates and potential to differentiate into three mesenchymal lineages (namely, osteogenic, adipogenic, and chondrogenic), to determine the best clone as the candidate cell source for further tissue engineering research.

We have recently reported the feasibility of using human DPSCs as bladder SMC progenitors for the regeneration of SMCs [20]. Although the capacity of DPSC differentiation into SMCs has been demonstrated, whether they can form a smooth muscle layer and its underlying molecular mechanisms remains largely unknown. The Wnt signaling pathway is an ancient and evolutionarily conserved pathway which orchestrates a range of biological processes, such as cell fate determination during embryonic development, cell proliferation, cell cycle arrest, differentiation, and apoptosis, as well as tissue homeostasis [21]. *β*-Catenin is a plasma membrane-associated protein that acts as an intracellular signaling transducer in the Wnt signaling pathway [22]. It has been demonstrated to induce the myogenic differentiation of rat MSCs through upregulation of myogenic regulatory factors [23]. Glycogen synthase kinase 3*β* (GSK3*β*) is a proline-directed serine-threonine kinase, and its phosphorylation appears to be a critical step in directing *β*-catenin to the nucleus [22]. Myogenic growth factors, including platelet-derived growth factor-homodimer polypeptide of B chain (PDGF-BB), transforming growth factor-*β*1 (TGF-*β*1), and vascular endothelial growth factor (VEGF), as well as hepatocyte growth factor (HGF), have been proven to play a vital role in SMC differentiation *in vivo* [24]. Therefore, the aim of this study is to analyse the mechanisms of the Wnt signaling pathway and the expression of myogenic growth factors involved in the regulation of differentiation of DPSCs toward bladder SMCs using the *in vitro* model we established before.

## 2. Materials and Methods

### 2.1. Human DPSC Clones and SMC Isolation

The pulp tissues were obtained from third molars (donors aged from 17 to 20 years) with the patient's informed consent and ethical approval by the South East Wales Research Ethics Committee of the National Research Ethics Service (permission number: 07/WESE04/84). The clonal populations of DPSCs were isolated using a fibronectin-based selection protocol as described previously [20, 25] after ethical approval and patient consent (permission number: 07/WESE04/84). Following 12 days of culture, single cell-derived clones were isolated using cloning rings and accutase digestion and then expanded. Three clones were selected, named as A11, B11, and A32. The level of population doublings (PDs) during expansion culture was monitored to measure the proliferation rate of the three clones [20]. Then, the three clones were induced to differentiate into three mesenchymal lineages (including osteogenic, adipogenic, and chondrogenic) in appropriate differentiation condition *in vitro* to compare their capacities of differentiation.

Human SMCs were obtained as reported previously from the bladder of patients who underwent open procedures for their bladder, after patient consent and ethical approval by the South East Wales Research Ethics Committee of the National Research Ethics Service (permission number: 07/WESE04/84) [20]. Briefly, bladder muscle tissue was minced into 1 × 1 mm pieces and digested in collagenase type IV enzyme (Sigma-Aldrich) for 30 minutes at 37°C. The digested muscle tissues were plated in Dulbecco's modified Eagle's medium (DMEM) with 10% FBS for establishing the primary culture.

### 2.2. Differentiation of Human DPSC Clone A32 and Wnt Pathway Inhibition Assay

Differentiation of the A32 was induced by using conditioned medium (CM) collected from bladder SMC culture, supplemented with transforming growth factor beta 1 (TGF-*β*1), as previously described [20].

The Wnt pathway was analysed by using the inhibitors including XAV939 (Wnt/*β*-catenin signaling inhibitor, Sigma), SB216763 (GSK-3 inhibitor, Sigma), and LiCl (GSK-3 inhibitor, Sigma), as previously described [26–28]. Briefly, cells were seeded into a 6-well plate. At 80% confluence of cells, the culturing medium was changed into the bladder SMC differentiation induction medium with the XAV939 (5 *μ*M), SB216763 (20 *μ*M), or LiCl (2.5 mM). An equivalent amount of dimethylsulfoxide was added to the control wells. After 14 days of incubation, the mRNA and protein levels were compared to the control group using qPCR and western blotting methods, respectively.

### 2.3. Immunocytochemistry

The cells were fixed with 4% PFA for 30 min and then incubated in 0.1% Triton X-100 for 10 min on ice and then blocked with bovine serum albumin (BSA) for 60 min at 37°C. After the blocking step, the cells were incubated with antimyosin (1 : 50), anti-*α*-SMA (1 : 100), and antidesmin (1 : 50) at 4°C, overnight; PBS was used as the negative control. The cells were then washed with PBS 3 times and incubated with anti-mouse IgG Alexa Fluor-488 or Alexa Fluor-594 secondary antibodies for 1 h at room temperature. The nuclei were counterstained with DAPI (VectorLabs). The protein expressions were observed with a fluorescent microscope and analysed with ImageJ software.

### 2.4. Flow Cytometry

The cells were washed and resuspended in PBS supplemented with 3% FBS that contained saturating concentrations (1 : 100 dilution) of the following reagents: FITC-conjugated anti-human monoclonal antibodies, anti-CD29-phycoerythrin (PE), anti-CD90-PE, anti-CD34-PE, anti-CD45-PE, anti-CD146-PE, or anti-STRO-1-allophycocyanin (APC) for 1 h at room temperature in the dark. As a negative control, PE- and APC-conjugated nonspecific mouse IgG1 were substituted for the primary antibodies. The cell suspensions were washed twice, resuspended in 3% FBS/PBS, and analysed with a flow cytometry cell-sorting Vantage cell sorter (Becton Dickinson). The data were analysed with a Mod-Fit 2.0 cell cycle analysis program (Becton Dickinson).

### 2.5. Real-Time Quantitative PCR (qPCR)

Total RNA was extracted from the cells using an RNeasy Mini Kit (QIAGEN) according to the manufacturer's directions. The total yield of RNA per extraction was calculated using a NanoVue spectrophotometer (GE Healthcare) to measure the absorbance at 260 nm. A260/A280 ratios of 1.9-2.1 indicated extraction of high-quality RNA. cDNA was synthesised with 2000 ng RNA using MMLV reverse transcriptase (Promega). For qPCR, three separate cDNA samples were used and each measured in triplicate. Target-specific primers (Table 1) were added to each cDNA sample together with Precision MasterMix with ROX and SYBR green (PrimerDesign). The PCR reaction was run by the ABI Prism fast 7500 qPCR system (Advanced Biosystems) under the following cycling conditions: an initial denaturation step of 95°C for 2 minutes followed by 40 cycles of 15 seconds denaturation (95°C) and 1 minute annealing/elongation at 60°C. The relative amount or fold change of the target gene expression was normalized relative to the level of D-glyceraldehyde-3-phosphate dehydrogenase (GAPDH) and relative to a control sample (noninduced cells).

### 2.6. Western Blot Analysis

The total protein was extracted from the cells with lysis buffer containing protease inhibitors (Roche, UK). The protein concentration was measured by a BCA-200 protein assay kit (Pierce, USA). Equal amounts of proteins were separated by 4-12% sodium dodecyl sulfate/polyacrylamide gel electrophoresis and transferred to a polyvinylidene fluoride (PVDF) membrane. The membrane was blocked in TRIS-buffered saline with Tween (TBST) containing 5% fat-free milk for 2 h and probed with primary antibodies, p-GSK3*β* (1 : 1000; Cell Signaling), t-GSK3*β* (1 : 1000; Cell Signaling), active *β*-catenin (1 : 1000; Cell Signaling), myosin (1 : 500; Sigma), *α*-SMA (1 : 500; Sigma), desmin (1 : 500, Sigma), and GAPDH (1 : 1000; Cell Signaling) overnight at 4°C and then incubated for 2 h with a horseradish-peroxidase-conjugated anti-mouse IgG antibody or anti-rabbit IgG diluted 1 : 2000 (Cell signaling). Protein bands were visualized on an X-ray film by using an enhanced chemiluminescence system (GE Healthcare, Buckinghamshire, UK). The relative protein expression intensities were quantified by densitometry using Quantity One analysis software.

### 2.7. Statistical Analysis

Each experiment was performed at least three times, unless otherwise indicated. Data are reported as the mean ± SD (standard deviation) deviation from three independent experiments. The significance of the differences between the experimental and the control groups was determined by using one-way analysis of variance; *P* < 0.05 indicated statistical significance.

## 3. Results

### 3.1. The Proliferation and Differentiation Ability of Three Clones of Human Dental Pulp Stem Cells (DPSCs) (A11, B11, and A32) and Characterization of A32

**Figure 1:**
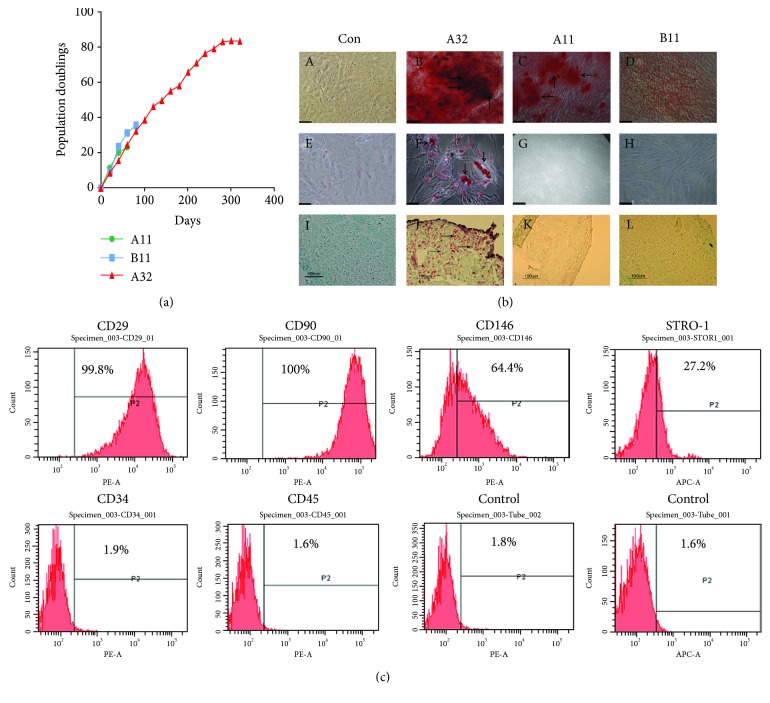
The ability of proliferation and differentiation analysis for three clones of human dental pulp stem cells (DPSCs) (A11, B11, and A32) and characterization of A32. Population doublings (PDs) of three clones (A11, B11, and A32) from different patients (a). The differentiating potential of the three clones into osteogenic (Alizarin Red staining) (b: B–D), adipogenic (Oil Red O staining) (b: F–H), and chondrogenic lineages (Safranin O staining) (b: J–L) when cultured in differentiation condition compared to control groups, respectively (b: A, C, and I). A32 had the potential to differentiate into osteogenic (b: B), adipogenic (b: F), and chondrogenic (b: J) lineages. A11 and B11 had the potential to differentiate into osteogenic lineages (b: C and D). Analysis of molecular surface antigen markers in A32 by flow cytometry (*P2*-positive zone of antigen) indicated that A32 was negative for CD34 and CD45, whereas it was positive for CD29 and CD90; of cells, 64.4% were CD146-positive and 27.2% STRO-1-positive (c). PE- and APC-conjugated nonspecific mouse IgG1 served as negative controls.

Dental pulp cells were isolated from pulp tissue of extracted third molars from patients. Three clones of cells that adhered to fibronectin were selected, noted as A11, B11, and A32. The proliferation rate and differentiation potential of the three clones were analysed. A32 demonstrated a high proliferation capacity extending beyond 80PDs, whilst the other two clones (A11 and B11) exhibited less than 36PDs (Figure 1(a)). Compared to A11 and B11 clones, A32 showed the best differentiation capacity into three mesenchymal lineages including osteogenic, adipogenic, and chondrogenic competency (Figure 1(b), B, F, J). The clone A32 was further characterized by flow cytometric analysis, which revealed that A32 was negative for CD34 and CD45. The culture population contained 99.8% CD29-positive cells, 100% CD90-positive cells, 64.4% CD146-positive cells, and 27.2% STRO-1-positive cells (Figure 1(c)).

### 3.2. The Smooth Muscle Layer-Like Phenotype Generated by the DPSC Clone A32 after SMC Induction

**Figure 2:**
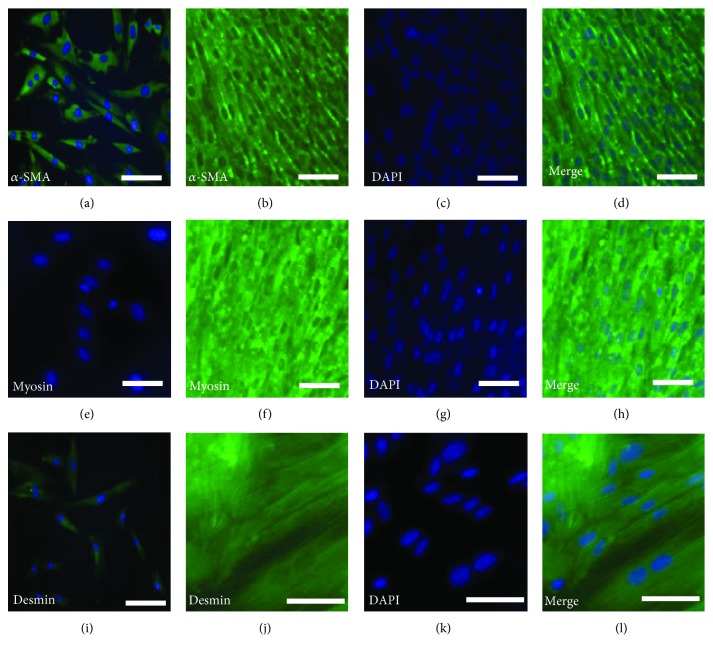
The smooth muscle layer-like phenotype generated by the dental pulp stem cell (DPSC) clone A32 after SMC induction. After a long period of differentiation (20 days), the induced A32 formed a monolayer and generated the smooth muscle-like phenotype which was positive for expression of the SMC markers, *α*-SMA (b–d), myosin (f–h), and desmin (j–l). Noninduced A32 as the negative control (a, e, i). The green staining indicates a positive result. Nuclei were stained with DAPI. Bars: 50 *μ*m.

Our previous study proved that the DPSC clone A32 displayed the potential to differentiate into SMCs by induction of CM from bladder SMCs in combination with TGF-*β*1. In this study, we evaluate whether DPSCs can generate a smooth muscle layer after an extended differentiation duration. Noninduced A32 were found to express *α*-SMA (Figure 2(a)) and desmin (Figure 2(i)) already, but none of these cells stained positive for myosin (Figure 2(e)). The induced cells from clone A32 formed a monolayer structure and also generated the smooth muscle-like phenotype which were shown to be positive for the SMC markers such as *α*-SMA (Figures 2(b)–2(d)), myosin (Figures 2(f)–2(h)), and desmin (Figures 2(j)–2(l)) after a longer period of differentiation (up to 20 days).

### 3.3. The Involvement of Wnt-Mediated GSK3*β*/*β*-Catenin in the SMC Differentiation from DPSC Clone A32

**Figure 3:**
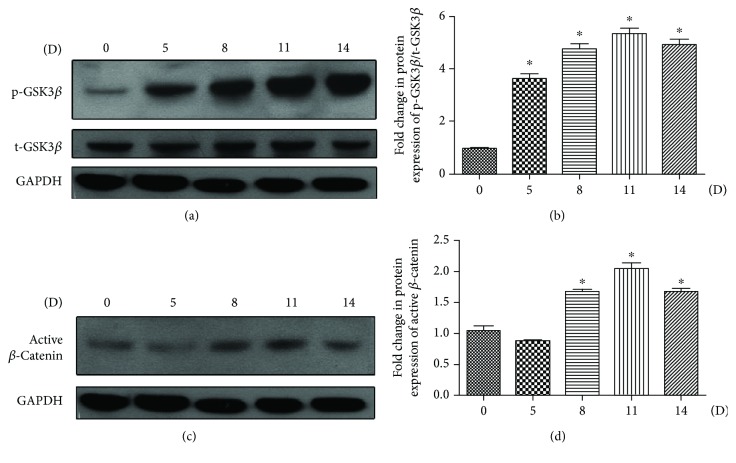
The involvement of Wnt-mediated GSK3*β*/*β*-catenin in SMC differentiation from DPSC clone A32. The A32 clone was induced according to the SMC induction protocol, 20% conditioned medium, and 2.5 ng/mL TGF-*β*1 for the indicated time (0 d, 5 d, 8 d, 11 d, and 14 d). The protein levels of p-GSK3*β*, t-GSK3*β*, and *β*-catenin were analysed with western blotting (a and c). The relative band intensities were determined by densitometry (b and d). Statistical analysis was performed by using one-way ANOVA. Date are shown as means ± SEM. ^∗^*P* < 0.05 when compared with the 0 d group.

The Wnt-GSK3*β*/*β*-catenin signaling pathway has been previously reported to play an important role in TGF-*β*1-induced MSC differentiation. To investigate whether this canonical Wnt signaling is also involved in the bladder SMC differentiation from DPSCs, we evaluated the protein level of phosphorylation GSK3*β* (p-GSK3*β*), total GSK3*β* (t-GSK3*β*), and active *β*-catenin in A32 in response to the induction of differentiation medium by western blotting. The protein level of p-GSK3*β* increased and maintained at the peak through 11 to 14 days (Figures 3(a) and 3(b)), while the protein level of t-GSK3*β* did not change (Figure 3(a)). As for the expression of active *β*-catenin, a significant upregulation was detected since day 8 of induction, which reached to the peak expression at day 11 (Figures 3(c) and 3(d)).

### 3.4. Inhibition of the Wnt-GSK3*β*/*β*-Catenin Signaling Pathway Prohibits the SMC-Specific Markers in the DPSC Clone A32 following SMC Induction

**Figure 4:**
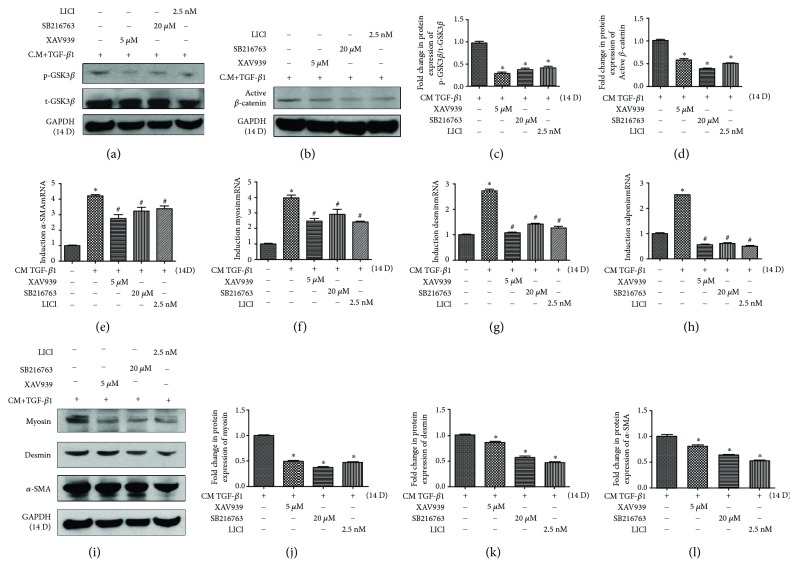
Inhibition of Wnt-GSK3*β*/*β*-catenin prohibits the SMC differentiation from DPSC clone A32. The A32 clone was induced following the SMC induction protocol with or without the Wnt/*β*-catenin-specific pharmacological inhibitor, XAV939, as well as the GSK3 inhibitors, SB216763 and LiCl, for 14 d. The mRNA expression of *α*-SMA, myosin, desmin, and calponin were analysed with qPCR (e–h); the protein levels of p-GSK3*β*, t-GSK3*β*, active *β*-catenin, *α*-SMA, myosin, and desmin were analysed with western blotting (a, b, and i). The relative band intensities were determined by densitometry (c, d, and j–l). Statistical analysis was performed by using one-way ANOVA. Date are shown as means ± SEM. ^∗^*P* < 0.05 when compared with the control group.

To further investigate whether canonical Wnt signaling is required for the SMC differentiation of cell clone A32, we assessed the SMC-specific markers, including *α*-SMA, myosin, and desmin, in the presence or absence of the Wnt/*β*-catenin-specific inhibitor, XAV939 and GSK3 inhibitors, SB216763, and LiCl. Pharmacological inhibition of Wnt and GSK3 significantly downregulated the protein expression of p-GSK3*β* (Figures 4(a) and 4(c)) and active *β*-catenin (Figures 4(b) and 4(d)) but had no effect on t-GSK3*β* (Figure 4(a)). The inhibition triggered a further reduction on the expression of SMC-specific markers including *α*-SMA, myosin, and desmin, at both gene and protein levels (Figures 4(e)–4(l)), compared to those of the non-inhibitor-treated control group of A32 following an SMC induction protocol for 14 days.

### 3.5. Growth Factor Analysis in SMC Differentiation from A32

**Figure 5:**
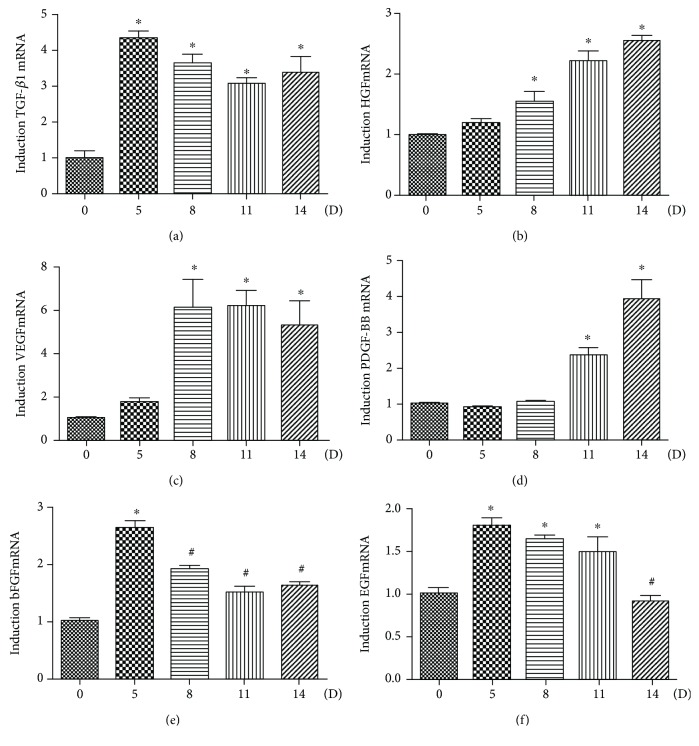
Growth factor analysis in SMC differentiation from DPSC clone A32. The A32 clone was induced following the SMC induction protocol for the indicated time (0 d, 5 d, 8 d, 11 d, and 14 d). The mRNA expressions of TGF-*β*1 (a), HGF (b), VEGF (c), PDGF-BB (d), bFGF (e), and EGF (f) were analysed with qPCR. Statistical analysis was performed by using one-way ANOVA. Date are shown as means ± SEM. ^∗^*P* < 0.05 when compared with the 0 d group.

The mRNA expression of growth factors, including bFGF, EGF, TGF-*β*1, HGF, VEGF, and PDGF-BB, in the process of SMC differentiation from DPSC clone A32 were detected by qPCR. The mRNA expression of TGF-*β*1 was notably increased through 5 to 14 days of differentiation (Figure 5(a)); the HGF and VEGF expression both increased since day 8 of differentiation and reached its peak level at day 14 (Figures 5(b) and 5(c)); the PDGF-BB expression appeared to go up after 11 days of differentiation and reached the highest level at day 14 (Figure 5(d)); the bFGF expression rose to its peak at day 5 but subsequently dropped with 14-day induction (Figure 5(e)); the EGF expression level was increased and maintained through 11 days of differentiation and then downregulated by day 14 (Figure 5(f)).

### 3.6. Wnt-GSK3*β*/*β*-Catenin Signaling Pathway Promotes the DPSC Clone A32 to Differentiate into Mature SMC via Regulating the Expression of Growth Factors

**Figure 6:**
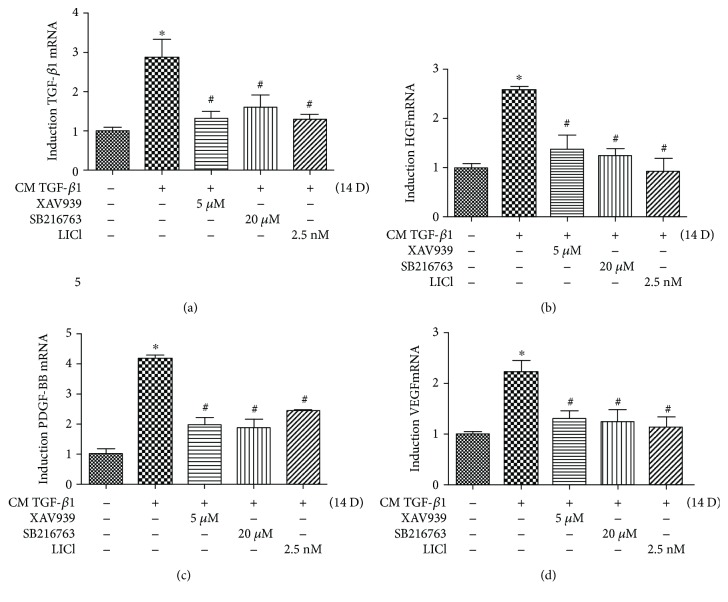
Wnt/GSK3*β*/*β*-catenin is involved in the process of SMC differentiation from the DPSC clone A32 through regulation of growth factors.The A32 clone was induced following the SMC induction protocol with or without the Wnt/*β*-catenin-specific inhibitor, XAV939, as well as the GSK3 inhibitors, SB216763 and LiCl, for 14 d. The mRNA expression of TGF-*β*1 (a), HGF (b), PDGF-BB (c), and VEGF (d) were analysed with qPCR. Statistical analysis was performed by using one-way ANOVA. Data are shown as means ± SEM. ^∗^*P* < 0.05 when compared with the control group.

We have demonstrated that Wnt-GSK3*β*/*β*-catenin signaling as well as several key growth factors was required in the process of human DPSCs differentiating into human bladder SMCs. To further confirm the involvement of Wnt signaling in the process of SMC differentiation from clone A32 by regulating the expression of growth factors, pharmacological inhibitors of the Wnt pathway were used to evaluate the change of gene expression of several key growth factors at the latter stage of SMC differentiation. After 14 days of differentiation, the gene expression of several growth factors including TGF-*β*1, HGF, PDGF-BB, HGF, and VEGF markedly upregulated (Figures 6(a)–6(d)), whilst either Wnt/*β*-catenin-specific inhibitor (XAV939) or GSK3 inhibitors (SB216763 and LiCl) significantly downregulated the expression levels of these growth factors (Figures 6(a)–6(d)), which suggests a promoting effect of Wnt-mediated GSK3*β*/*β*-catenin signaling on SMC differentiation of clone A32 by regulating the release of growth factors including TGF-*β*1, HGF, PDGF-BB, and VEGF.

## 4. Discussion

SMC-based tissue engineering provides a potential therapy for SMC pathology, including cardiovascular diseases, gastrointestinal diseases, urinary incontinence, and bladder dysfunction [29–31]. A reliable cell source of healthy SMCs that can be easily obtained and safely expanded plays a vital role in promoting the progress of smooth muscle tissue engineering. DPSCs have demonstrated the advantages of easy access with the least invasive procedures and little inherent immunogenicity but without any ethical issues, which make them a promising cell population for developing tissue engineering and regenerative medicine [16]. In order to use DPSCs for clinical therapy, *in vitro* expansion and differentiation ability should be taken into account for the success of clinical applications in tissue engineering. In this study, three clones of DPSCs from two patients were isolated, which were named as A11 and A32 (from patient A) and B11 (from patient B). Investigation of the proliferation rate using PDs as an indicator demonstrated that two of the clones (A11 and B11) showed less than 36PDs and senesced within 85 days, while A32 had a high proliferation rate with over 85PDs that only started senescing after more than 300 days. Compared with the previous studies about the analysis of proliferation rate of MSCs from bone marrow [32–34], stem cell clone A32 displayed a better proliferation capacity, giving this clone a potential advantage. It has been reported that only a small percentage of the overall clonal population of DPSCs, which is less than 5% of the total population, has the ability to differentiate into three mesenchymal lineages, including osteoblast, chondrocytes, and adipocytes [35–37], whilst our data demonstrated that all the three clones (A32, A11, and B11) displayed good ability to differentiate into the osteogenic phenotype, among which A32 was able to differentiate into three different lineages. This result is consistent with data presented by Halleux et al., in which 24 designated clones differentiated into osteogenic lines, while 17/24 and 18/24 clones differentiated into chondrogenic and adipogenic lineages, respectively [35]. The niche of these located clones and the different stages of their development may be accountable for their different growth kinetic profiles and differentiation abilities. Therefore, DPSC clone A32 which has the best proliferation and differentiation ability was selected for further bladder tissue engineering research. We characterized clone A32 by using flow cytometric analysis. The cell populations were positive for a range of mesenchymal stem cell markers including CD29, CD90, CD146, and STRO-1 but negative for hematopoietic stem cell markers, such as CD34 and CD45, which demonstrated that the clone A32 is sourced from the mesenchymal stem cells, not hematopoietic stem cells.

The urinary bladder wall is mainly composed of the Detrusor smooth muscle layer which is made of smooth muscle fibers arranged in spiral, longitudinal, and circular bundles [38]. The Detrusor smooth muscle is mainly responsible for storing urine under low pressure and contraction for voiding, lined by a layer of transitional cells that provide a barrier to absorption. Therefore, it is a crucial step to regenerate the Detrusor smooth muscle layer as a tissue-specific effort for the tissue engineering of the urinary bladder. A previous study in our lab has demonstrated that *α*-SMA and desmin were already present in noninduced human DPSC clone A32 at a low basal level, which indicates A32 may be more suitable to be induced into SMCs for bladder tissue regeneration. Additionally, this clone has the potential to differentiate into bladder SMC by the CM from cultured bladder SMC in combination with TGF-*β*1 [20]. In this study, we further proved that A32 could generate a smooth muscle layer-like phenotype after an extended differentiation duration. Further functional analysis would be required using 3D cell culture by seeding the DPSC-derived smooth muscle layer-like structure into synthetic bladder composites and transplanting them into nude rats which underwent removal of half the bladder, in order to evaluate its regeneration capacity of the Detrusor smooth cells *in vivo*.

Canonical Wnt/*β*-catenin signaling plays a primary role in the regulation of proliferation and differentiation of stem cells [21, 39]. GSK3 exists as two highly homologous isoforms encoded by distinct genes known as GSK3*α* and GSK3*β*. GSK3*β* originally isolated from muscles as a kinase promotes glycogen assimilation by phosphorylating and inactivating glycogen synthase. It is also involved in the maintenance and plasticity of the skeletal muscle mass, as well as playing an important role in skeletal muscle atrophy *in vivo* [40, 41]*. β*-Catenin is a multifunctional protein which is located in both the nucleus and cytoplasm. It leads to myogenic specification and prevents adipogenic differentiation in adult stem cells [42–44]. GSK3*β* and *β*-catenin are key factors in the canonical Wnt pathway. Activation of the Wnt signaling promotes the stabilization and accumulation of *β*-catenin in the cytoplasm by triggering GSK3*β* phosphorylation which prevents subsequent GSK3*β*-mediated *β*-catenin phosphorylation in association with Axin and adenomatous polyposis coli (APC) [21]. The stabilized *β*-catenin enters the nucleus and induces activation of target genes by binding with members of the T-cell factor (TCF) and lymphoid enhancer factor (LEF) transcription factor family [45]. It has been reported that Wnt-GSK3*β*/*β*-catenin contributes to the progressive nature of smooth muscle tissue remodelling [22] and also acts as an indispensable regulator for myogenesis in embryogenesis and postnatal muscle regeneration [46], as well as inducing myogenic differentiation in stem cells during muscle regeneration [42]. In this study, we found that Wnt-GSK3*β*/*β*-catenin signaling is involved in regulating the process of bladder SMC differentiation from human DPSCs by the SMC induction protocol. To confirm the effect of Wnt/*β*-catenin signaling and GSK3*β* on SMC differentiation, two types of pharmacological inhibitors were used. One is XAV939 which is a tankyrase inhibitor for inhibiting Wnt/*β*-catenin signaling [28]. The others are SB216763 and LiCl which block GSK3 activation [26, 27]. SB216763 is a small molecule that competes with ATP and potently inhibits the activity of *α* and *β* isozymes of GSK3. Considering the high degree of sequence similarity, GSK3*α* and GSK3*β* share some similar functions. For example, the single loss of either GSK3*α* or GSK3*β* in mouse embryonic stem cells (ESC) did not negatively alter Wnt/*β*-catenin signaling, whereas GSK3*α*/*β* double knockout ESCs displayed hyperactivated Wnt/*β*-catenin signaling, resulting in dramatically skewed cell differentiation [47]. Nevertheless, the two isoforms were shown to have opposite effects on the transcriptional activation of certain transcription factors [48]. GSK3*α* or GSK3*β* plays distinct roles in cardiomyocyte differentiation and cardiovascular development in mice [49–51]. Therefore, it appears that GSK3*α* and GSK3*β* have both common and nonoverlapping cellular functions, largely depending on the physiological context and the cell type studied. It has been reported GSK3*β* protein expression in human and mouse muscles was found to be three to four times higher than GSK3*α*, suggesting that GSK3*β*, rather than GSK3*α*, may be the predominant GSK3 isoform in the muscle [6]. Two types of inhibitors significantly suppressed the activation of p-GSK3*β* as well as active *β*-catenin and also downregulated the expression of SMC markers (especially myosin which is only expressed in contractile SMCs) at the latter stage of differentiation at both mRNA and protein levels. It suggests that Wnt-GSK3*β*/*β*-catenin signaling promotes the human DPSCs to differentiate into bladder SMCs. Further analysis would be required by downregulation of GSK3*α* or GSK3*β* to investigate the role of GSK3*α* in regulating the process of DPSC differentiation into SMC.

Previous studies have identified that a variety of stem cells, including those from the embryo [52, 53], bone morrow [54], adipose tissue [55], and dental pulp tissue [20] can be induced to differentiate into bladder SMCs by using differentiation agents, such as CM from SMC culture in addition to myogenic growth factors, or by indirectly coculturing with the target cells. The CM or indirect coculture system contains cytokine growth factors secreted by the target cells that can be used to induce cell differentiation [56]. Here, we showed that several growth factors, including TGF-*β*1, HGF, VEGF, PDGF-BB, bFGF, and EGF, play different roles in the process of SMC differentiation. It has been demonstrated that TGF-*β*1 evokes an important signal that induces expression of vascular SMC markers in a range of nonsmooth muscle precursor cell types, including multipotent embryonic fibroblast [57], neural crest cells [58], and MSCs [54]. We here found that the expression of TGF-*β*1 upregulated during the whole process of SMC differentiation, indicating that TGF-*β*1 also plays an important role in the bladder SMC differentiation from DPSCs. It has been shown previously that an increased expression of bFGF was associated consistently with SMC proliferation of the neointima formation [59] and atheromatous lesions [60] *in vivo*. Additionally, experimental inhibition of bFGF activation leads to the SMC marker expression in the model of BMMSC differentiation into SMC [8]. As one of the most important mitogenic growth factors, bFGF is known to promote proliferation of marrow cells [61], which is consistent with our data, because the phenomenon of DPSC proliferation was observed when the expression of bFGF was upregulated during 5 days of SMC differentiation (data not shown). PDGF-BB, which is previously associated with proliferation and differentiation of SMCs [62, 63], was upregulated after 11 days of differentiation in our study, indicating that this growth factor is mainly for promoting bladder SMC differentiation from DPSCs. As for the expression of myogenic growth factors, such as HGF and VEGF, they were both upregulated after 8 days of differentiation, which is consistent with the previous study of BMMSC differentiation into bladder SMCs [54]. The expression levels of the growth factors, including TGF-*β*1, HGF, VEGF, and PDGF-BB, were all upregulated at 14 days of differentiation, indicating that these growth factors may induce the maturity of SMCs at the latter stage of bladder SMC differentiation from DPSCs. In order to further investigate whether Wnt-GSK3*β*/*β*-catenin signaling is involved in the mature process of SMC differentiation, we evaluated the expression of the growth factors above with or without two types of inhibitors at a later stage of SMC differentiation. We noticed that the expression of these growth factors was significantly downregulated with the Wnt/*β*-catenin inhibitor and GSK3*β* inhibitors at that stage, indicating that the Wnt-GSK3*β*/*β*-catenin signaling promotes the human DPSC differentiation into mature bladder SMCs by regulating the expression of these myogenic growth factors.

**Figure 7:**
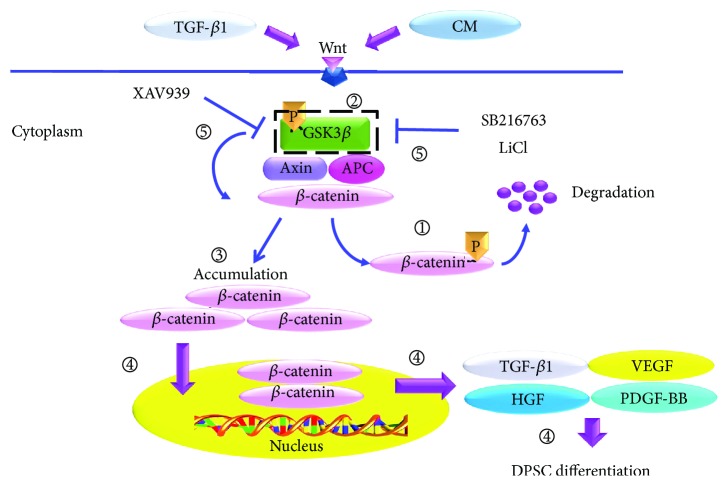
Wnt/GSK3*β*/*β*-catenin activation. 1: in the absence of canonical Wnt signaling, *β*-catenin is complexed with GSK3*β*, APC, and Axin, which facilitate the phosphorylation and subsequent degradation of *β*-catenin. 2: activation of canonical Wnt signaling by the growth factor, TGF-*β*1, and CM from SMC triggers phosphorylation of GSK3*β*, thereby disrupting the APC/Axin/GSK3*β*/*β*-catenin complex. 3: *β*-catenin which dissociates from the APC/Axin/GSK3*β* complex escapes from degradation and accumulates in the cytoplasm. 4: the stabilization and accumulation of *β*-catenin translocates into the nucleus and enhances the expression of growth factors, including TGF-*β*1, HGF, VEGF, and PDGF-BB, thus promoting the SMC differentiation from the DPSC clone A32. 5: Wnt/*β*-catenin signaling inhibitor, XAV939, and GSK3 inhibitors, SB216763 and LiCl, were used to block the Wnt/GSK3*β*/*β*-catenin signaling.

As summarized in Figure 7, we hypothesize that the canonical Wnt which is activated by CM from SMCs in combination with TGF-*β*1 triggers phosphorylation of GSK3*β*, thus disrupting the APC/Axin/GSK3*β*/*β*-catenin complex. The stabilization and accumulation of *β*-catenin in cytoplasm translocates into the nucleus and enhances the expression of growth factors, including TGF-*β*1, HGF, VEGF, and PDGF-BB, which subsequently promote the SMC differentiation from DPSCs, for instance, clone A32.

## 5. Conclusion

In this study we characterized one of our DPSC clones (clone A32) with the best proliferation and differentiation abilities and demonstrated that the clone A32 possesses a potential to differentiate into a bladder smooth muscle cell layer-like phenotype *in vitro* using the bladder SMC induction protocol we established before. Additionally, we have found that the Wnt-GSK3*β*/*β*-catenin signaling is involved in SMC differentiation. Several growth factors (including TGF-*β*1, HGF, VEGF, PDGF-BB, and bFGF) regulated by Wnt-GSK3*β*/*β*-catenin signaling promote the proliferation/differentiation during the process of bladder SMC differentiation from human DPSCs.

## Figures and Tables

**Table 1:** 

Genes	Forward and reverse primers	Accession number
GAPDH	5′-GCACCGTCAAGGCTGAGAAC-3′ 5′-TGGTGAAGACGCCAGTGGA-3′	NM_002046.3
*α*-SMA (ACTA2)	5′-CCGGTTGGCCTTGGGGTTCAGGGGTGCC-3′ 5′-TCTCTCCAACCGGGGTCCCCCCTCCAGCG-3′	NM_001141945.1
Myosin (MYH11)	5′-AAGAAAGACACAAGTATCACGGGAGAGC-3′ 5′-TGTCACATTAATTCCCATGAGGTGGCAA-3′	NM_001040113.1
Desmin	5′-CACCATGAGCCAGGCCTACTCGTCCA-3′ 5′-GGCAGCCAAATTGTTCTCTGCTTCTTCC-3′	NM_001927.3
Calponin	5′-GGCTCCGTGAAGAAGATCAATGAGTCAA-3′ 5′-CCCTAGGCGGAATTGTAGTAGTTGTGTG-3′	NM_001299.4
TGF-*β*1	5′-ATGCCGCCCTCCGGGCTGCGG-3′ 5′-CAGCTGCACTTGCAGGAGCGC-3′	NM_000660
HGF	5′-TACAGGGGCACTGTCAATACC-3′ 5′-GGATACTGAGAATCCCAACGC-3′	NM_000601.4
VEGF	5′-ACGTACTTGCAGATGTGACAAG-3′ 5′-GTGGCGGCCGCTCTA-3′	NM_001025366.2
PDGF-BB	5′-ATGAATCGCTGCTGGGCGCTC-3′ 5′-CTAGGCTCCAAGGGTCTCCTTC-3′	NM_002608.3
bFGF	5′-ACGGGGTCCGGGAGAAGAGC-3′ 5′-TGCCCAGTTCGTTTCAGTGCCA-3′	NM_002006.4
EGF	5′-CTTGTCATGCTGCTCCTCCT-3′ 5′-GAGGGCATATGAAAGCTTCG-3′	NM_001963.4

## Data Availability

The data used to support the findings of this study are included within the article.
